# Exploring different stroke populations’ information needs: a cross-sectional study in England

**DOI:** 10.1186/s13690-024-01289-2

**Published:** 2024-05-06

**Authors:** Allam Harfoush, Kausik Chatterjee, Elizabeth Deery, Hanady Hamdallah

**Affiliations:** 1 Chester Medical School, The Faculty of Medicine and Life Sciences, University of Chester, Chester, UK; 2 The Countess of Chester Hospital, Liverpool Road, Chester, UK; 3 The School of Sport, Ulster University, York St, Belfast, UK

**Keywords:** Prevention, Information, Education, Needs, Priorities, Stroke, Survivors

## Abstract

**Background:**

While tailored information might have the potential to motivate stroke survivors to make essential lifestyle changes and improve long-term outcomes, how this varies among different stroke populations is not yet fully understood.

**Method:**

From November 2022 to May 2023, stroke survivors in the UK, who were clinically stable, participated in a community-based, descriptive cross-sectional study. Participants rated several information themes on a Likert scale from one to five, indicating the relevance of each information group to them. Data were analysed using Wilcoxon and chi-squared tests on SPSS. Descriptive statistics were employed for examining the preferred information delivery method, timing, personnel, and frequency.

**Results:**

Seventy survivors, with an average age of 67 ± 19 (61% males), were recruited. Survivors emphasised the importance of symptoms, risk factors, and recovery information during hospital stay, while medication and lifestyle change information were more significant in the community. Subgroup analysis revealed distinct patterns: First-time stroke survivors highlighted the importance of social and financial support (acute phase median Likert score 3, chronic phase median Likert score 4; *p* < 0.01), while those with prior strokes emphasised information on driving and working after stroke (acute phase median Likert score 4, chronic phase median Likert score 3; *p* < 0.05). Survivors recruited after six months of stroke prioritised knowledge of carer support in the community (acute phase median Likert score 3.5, chronic phase median Likert score 4; *p* < 0.01).

**Conclusion:**

Survivors’ information needs differ depending on factors such as the recovery phase, type of stroke, time since diagnosis, and the presence of a previous stroke. Considering these factors is essential when developing or providing information to stroke survivors.

**Table:** 

Text box 1: Contributions to the literature
• This report uncovers new insights into how different groups of stroke survivors have unique information needs.
• We noticed a pattern in how survivors seek information about strokes, which is in line with theories on how individuals adapt socially following life-threatening events.
• When providing information to stroke survivors, it’s important to consider five key factors: the type of information, how it’s delivered, when it’s delivered, how often it’s delivered, and who delivers it.

## Introduction

It is estimated that adhering to necessary long-term management and healthy behaviour changes could prevent more than 50% of strokes [1]. However, the patient’s willpower and beliefs, together with their personal, social, and economic status would often influence these modifications [2, 3]. Providing relevant information about the benefits of leading a healthy lifestyle and the consequences of not making changes is essential [4]. Hence, the concept of patients’ information needs emerged to deliver pertinent information, motivating patients to adopt necessary healthy behaviour [5].

In stroke, the needed information vary with time due to the fluctuating emotional status experienced postdiagnosis [6, 7]. Therefore, considering the variation in stroke survivors’ information needs throughout their recovery is important. Some studies have shown differences in what survivors’ information prioritises, such as rehabilitation and preventing recurrence in the acute phase, while focusing on financial support in the long term [8–11]. However, the literature lacks a thorough exploration of the impact of time on stroke information needs and how this differs across various stroke populations. This study aims to investigate stroke survivors’ information needs during different recovery stages and evaluate appropriate approaches to deliver them.

## Methods

This study was descriptive, cross-sectional, observational research utilising a semistructured questionnaire (online supplement 1) and was approved by the University Ethics Committee (1901-22-AH-CMS) in October 2022. Recruitment took place from November 2022 to May 2023.

The questionnaire’s development began with a literature search to collect survivor-expressed information needs. Fourteen publications were reviewed, identifying 85 distinct information needs (online supplement 2). Using a pile-sorting method [12], these needs were categorised into ten groups. The categories were incorporated into our questionnaire, where participants rated these ten groups on a Likert scale from 1 to 5 based on the relevance of each group to them, covering two recovery phases: during hospital stay and in the community. Additionally, the questionnaire explored participants’ preferences for information delivery methods, desired healthcare professionals (HCPs) responsible for providing information, timing of delivery, and frequency of information dissemination. Open-ended questions were included to further explore participants’ views.

The first draft of the questionnaire underwent review by a stroke consultant and a stroke survivor focus group comprising 30 stroke survivors and carers. Their feedback was used to refine the questionnaire. Subsequently, pilot testing was conducted, involving nine stroke survivors identified from online stroke support groups in the UK, resulting in slight changes in the final version.

The study recruited participants who had experienced either a stroke (ischaemic or haemorrhagic) or a transient ischaemic attack (TIA), were currently living in the UK, and were clinically stable. Exclusions comprised individuals under 18 years old, those unable to communicate effectively in English, and those lacking mental capacity.

After completing the pilot testing phase, recruitment primarily occurred through in-person methods, utilising a paper-based questionnaire. Participants eligibility was determined by the stroke consultant from the stroke outpatient clinic at the Countess of Chester Hospital and the stroke rehabilitation centre at Ellesmere Port Hospital in northwest England, who provided information about the questionnaire, along with a poster featuring a quick response code for accessing the consent form and the questionnaire. Interested participants filled out the questionnaire anonymously. Additionally, suitable participants were invited to participate in the study through online UK-based stroke survivors’ support groups. The questionnaire included screening questions at the beginning to assess the eligibility of individuals identified online.

SPSS 26 was utilised for quantitative analysis. Initially, descriptive statistics were conducted. Subsequently, the normality of the independent variables distribution (the information needs Likert scale) guided the selection of statistical tests. The median of the Likert scale, ranging from one to five, was calculated and compared for each information group. For subgroup analysis, data was divided using SPSS into the relevant subgroups and the Likert scale median was compared before and after discharge using the Wilcoxon test. *P* values below 0.05 were deemed significant. The relative importance index (RII) for each IN category was calculated. The RII is used to weigh the different factors based on their Likert score value [13], and it is calculated using the equation: Σ𝑤/𝐴𝑁 = 5𝑛5 + 4𝑛4 + 3𝑛3 + 2𝑛2 + 1𝑛1 / 5𝑁, where n5 represents the number of participants who chose “extremely important” for one option (n4, n3, n2, n1 follow the same pattern) and N represents the total number of participants [14]. The RII has a range from 0 to 1, and a rank could be generated based on each category value. Missing data were excluded from the analysis.

Content analysis was conducted to analyse the qualitative data [15]. Responses were reviewed multiple times, coded, and organised into themes. The first author (A.H) and the third author (L.D) independently followed the same procedure to perform the analysis. A consensus meeting was held in June 2023 to finalise the outcomes.

## Results

The study included 70 participants with a median age of 67 ± 19 years, among whom 61.4% were male. Ischaemic stroke cases comprised 54.3% of the sample, with the majority (74.3%) being first-time stroke survivors. Additionally, 62.9% of participants had experienced their stroke within the last six months. Table 1 presents an overview of the baseline characteristics.

**Table 1:** Characteristics of study participants between November 2022 and May 2023 (*N* = 70)

Variable	Subgroup	N (%)
Age (years)	Less than 65	29 (41.4)
	More than 65	41 (58.6)
Type of stroke	Stroke (ischaemic and haemorrhagic)	46 (65.7)
	TIA	24 (34.3)
Gender	Male	43 (61.4)
	Female	27 (38.6)
Educational level	GCSE grades	46 (65.7)
	Above-GCSE grades	24 (34.3)
Time since stroke diagnosis	Less than 6 months	44 (62.9)
	More than 6 months	26 (37.1)
Recurrence	First-time stroke	52 (74.3)
	Previous stroke	18 (25.7)

N: number; TIA: transient ischemic attack; GCSE: General Certificate of Secondary Education

Statistically significant differences were noted between recovery phases in terms of Information needs. During the hospital stay, the emphasis was on understanding risk factors and the anticipated recovery. However, upon transitioning to the community, the focus shifted to knowledge about medications, access to carer and social support, financial aid, and insurance with a *p* value < 0.05, as depicted in Table 2.

**Table 2:** The median of information needs in each stage assessed using a five-point Likert scale on each of the ten extracted categories between November 2022 and May 2023 (*N* = 70)

**Information need group**	**During hospitalisation**	**In the community**	**Significance**
	**Median** **(Min, Max)**	**Median** **(Min, Max)**	
Stroke symptoms and how to respond to them	5 (1,5)	4 (2,5)	**NS**
Medications and follow-up	**4** (1,5)	**5** (2,5)	**
Stroke risk factors and how to control them	**5** (1,5)	**4.5** (2,5)	**
Recommended lifestyle modifications	4 (2,5)	4 (2,5)	**NS**
Predicted recovery and consequences	**5** (2,5)	**4** (2,5)	**
Working and driving after a stroke	4 (1,5)	4 (1,5)	**NS**
Social support, financial assistance, and insurance	**3** (1,5)	**4** (1,5)	**
Where and how to get more information	4 (1,5)	4 (1,5)	**NS**
The rights and responsibilities	3 (1,5)	3 (1,5)	**NS**
The availability of carer’s support	**4** (1,5)	**4** (1,5)	*

Mn: Minimum; Mx: Maximum; *: *P* ≤ 0.05; **: *P* ≤ 0.01; NS: *P* > 0.05

**Fig. 1:**
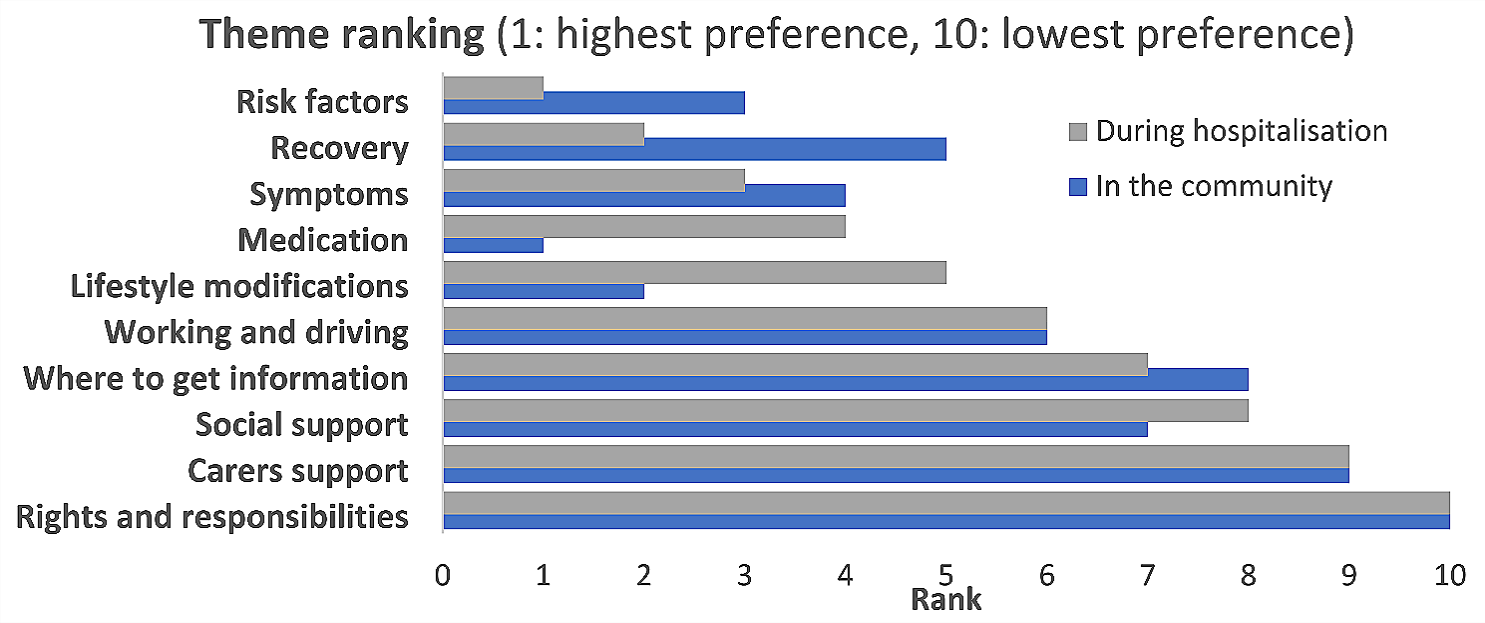
Rank of the needed information themes before and after discharge using the relative importance index (RII)

The ranking of the information needs based on the RII values showed that knowledge of risk factors, recovery, and symptoms were the top three information needs during hospitalisation, respectively. However, knowledge of medications, lifestyle modifications, and risk factors became the top three in the community, as shown in Fig. 1.

Subgroup analysis was conducted by dividing the dataset based on the underlying diagnosis (stroke or TIA), time since diagnosis (< 6 months or > 6 months), and recurrence (first time or previous stroke). The median of the Likert scale (ranging from one to five) for each category was compared in both the chronic and acute phases. Statistically significant differences among subgroups were summarised in Table 3. Significant differences were found based on the underlying diagnosis. Stroke survivors prioritised knowledge about recovery during hospitalisation, whereas medication and social support information took precedence in the community setting. Those recruited six months postdiagnosis emphasised the importance of understanding social, financial, and carer support after discharge. Additionally, individuals with a history of stroke highlighted the significance of information regarding driving and working poststroke in acute settings.

**Table 3:** Statistically significant differences in information needs among participants’ subgroups at each stage, assessed using a five-point Likert scale between November 2022 and May 2023

Subgroup	Information needs	Hospitalisation	In the community
Stroke	Medications		**
	Recovery	*	
	Social support		**
TIA	Risk factors	*	
< 6 months since diagnosis	Medications		*
	Risk factors	*	
	Recovery	**	
> 6 months since diagnosis	Social support		*
	Carer support		**
First-time stroke	Risk factors	**	
	Recovery	*	
	Social support		*
Previous stroke	Medication		*
	Recovery	*	
	Working and driving	*	

The grey squares are the distribution of statistically important differences based on the stroke stage

*: *P* ≤ 0.05; **: *P* ≤ 0.01

Participants reported that in-person discussions were the most preferred method of information delivery (74% of the sample), followed by written materials (50%) and the Internet (24%). Surprisingly, only 41.4% of respondents reported being offered take-home written materials before discharge. The most preferred timing for information delivery was during the rehabilitation phase, selected by 53% of the sample. Specialist neurologists were the top choice for discussing stroke-related information (58.5%), followed by general practitioners (GPs) and nursing staff (preferred by 43% and 30%, respectively). Interestingly, respondents indicated in open-ended questions the important role of physiotherapists in answering their questions about the different aspects of stroke during physiotherapy sessions. A majority of stroke survivors (61.4%) expressed the desire for multiple discussions about stroke information, whether it occurs every three, six, or twelve months.

Finally, in open-ended questions regarding the design of written materials, participants most frequently suggested including rehabilitation techniques and exercises. Other suggestions involved adding pictures, using larger font sizes, and presenting the content in an optimistic way, as outlined in Table 4.

**Table 4:** The qualitative analysis of the participants’ recommendations on the design of stroke-related written material between May 2022 and November 2023 (*N* = 30)

Code	Themes
• Pictorial information	Structure
• Large font	
• Relevant to all ages	
• Offered online	
• Simple and concise	
• Organised in a step-by-step way	
• Include rehabilitation techniques and home exercises	Content
• Be optimistic and include positivities	
• Focus on symptoms	
• Include the dos and don’ts after a stroke	
• Include how to cope with life after a stroke	
• Focus on lifestyle changes	

## Discussion

Understanding stroke survivors’ information needs throughout their recovery is vital to ensure active involvement with the management plan, especially since newly diagnosed survivors frequently express their limited understanding of the condition [16]. In our analysis, different information needs emerged across recovery phases. During hospitalisation, participants focused on risk factors and recovery information, while in the community phase, participants prioritised medications, social support, finances, and insurance. There were several differences between the needs during hospitalisation and after discharge for stroke survivors, where knowing the future risk and potential functional restoration was far more important in the hospitalisation phase, shifting to the knowledge of daily medications and available support in the community. A similar pattern was observed in previous studies [7, 8]. This difference is consistent with the theory of cognitive adaptation to life-threatening events [17], where initially there is a focus on understanding the event’s causes, its impact, and the likelihood of recurrence. This emphasis transitions to concerns about self-adjustment and managing daily life during the long-term phase.

Differences based on stroke type (stroke or TIA) may stem from the complexities of stroke recovery, often involving functional complications that necessitate long-term support [18]. Individuals recovering from stroke express concerns about available support, contrasting the potential for full recovery often observed in TIA survivors. Time since stroke diagnosis also influenced survivors’ information needs, as newly diagnosed individuals inquired about risk factors and recovery due to uncertainties surrounding long-term complications [19]. Those with over a 6-month diagnosis focused on themes related to available support, considering the potential decline in function and quality of life in the long term after stroke [20]. Recurrence further shaped concerns, with first-time stroke survivors focusing on preventing future episodes, while those with previous strokes emphasised moving forward, including aspects such as working and driving after stroke. These findings resonate with the theory of psychological adjustment in long-term diseases, progressing from initial stress and uncertainty about illness to eventual acceptance and emotional equilibrium [21].

The preferred method for delivering information involved a combination of verbal and written materials. Verbal communication allows for personalised information sharing, while written materials offer the advantage of revisiting information after discharge. Our sample suggested developing pictorial materials with large prints, which was recommended in the literature to aid information retention in the long term [22, 23]. Additionally, our study indicated an optimistic approach to presenting information, considering that some stroke survivors experienced despair and helplessness with current stroke-related written materials [24]. Participants proposed delaying information delivery until the rehabilitation phase, which acknowledges the challenge of retaining information in the early stages poststroke [25].

Specialist neurologists, GPs, and nurses emerged as the preferred healthcare professionals for discussing stroke-related matters. Although physiotherapists were not among the most frequently selected HCPs, their role in information-sharing is important due to the time they spend facilitating and applying rehabilitation techniques. This suggests a broader involvement in survivors’ education beyond merely performing exercise therapy. Finally, reiterating information was deemed necessary as survivors’ information needs evolve with time.

However, this study had limitations. Firstly, recruitment from a specific area will limit the generalisability of the outcomes, as other local and international facilities have different approaches and contact points from the one shown in our study. Secondly, the exclusion of participants with cognitive impairment. Conducting in-depth interviews might have offered deeper insights into the reasons behind the subgroup differences.

**Fig. 2:**
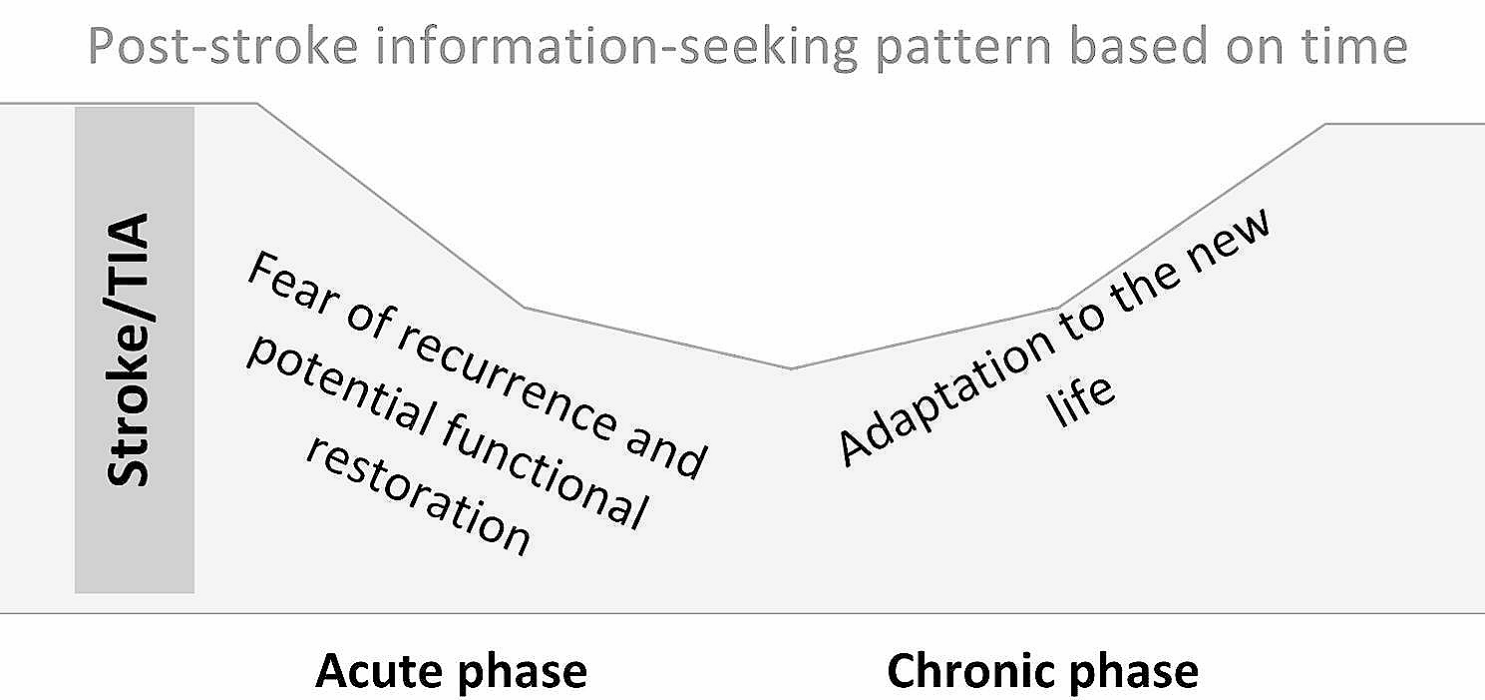
Stroke survivors’ information-seeking pattern

To conclude, post-stroke information-seeking displays two discernible patterns: during the acute phase, the focus is on reducing stroke recurrence and restoring function. However, in the chronic phase, attention shifts towards adapting to the new life poststroke, as outlined in Fig. 2. This could help healthcare professionals identify the most appropriate information based on the recovery phase, type, and time elapsed since diagnosis.

Accordingly, an optimal approach for stroke survivors involves tailoring information delivery based on these distinct patterns while considering the different stroke populations and emphasising suitable delivery methods and timing. Such an approach could enable healthcare providers to empower survivors to manage their condition more effectively. Further research is needed to investigate whether providing this tailored information could improve stroke survivors’ long-term outcomes.

## Data Availability

The data supporting the findings of this study is available from the corresponding author upon reasonable request.
